# The MHC-II transactivator CIITA inhibits Tat function and HIV-1 replication in human myeloid cells

**DOI:** 10.1186/s12967-016-0853-5

**Published:** 2016-04-18

**Authors:** Greta Forlani, Filippo Turrini, Silvia Ghezzi, Alessandra Tedeschi, Guido Poli, Roberto S. Accolla, Giovanna Tosi

**Affiliations:** Department of Surgical and Morphological Sciences, University of Insubria, Varese, Italy; Viral Pathogens and Biosafety Unit San Raffaele Scientific Institute, Milan, Italy; AIDS Immunopathogenesis Unit, San Raffaele Scientific Institute, Milan, Italy

**Keywords:** HIV-1 replication, Restriction factors, CIITA, TRIM22, U937 cells

## Abstract

**Background:**

We previously demonstrated that the HLA class II transactivator CIITA inhibits HIV-1 replication in T cells by competing with the viral transactivator Tat for the binding to Cyclin T1 subunit of the P-TEFb complex. Here, we analyzed the anti-viral function of CIITA in myeloid cells, another relevant HIV-1 target cell type. We sinvestigated clones of the U937 promonocytic cell line, either permissive (*Plus*) or non-permissive (*Minus*) to HIV-1 replication. This different phenotype has been associated with the expression of TRIM22 in U937 *Minus* but not in *Plus* cells.

**Methods:**

U937 *Plus* cells stably expressing CIITA were generated and HLA-II positive clones were selected by cell sorting and cloning. HLA and CIITA proteins were analyzed by cytofluorometry and western blotting, respectively. HLA-II DR and CIITA mRNAs were quantified by qRT-PCR. Tat-dependent transactivation was assessed by performing the HIV-1 LTR luciferase gene reporter assay. Cells were infected with HIV-1 and viral replication was evaluated by measuring the RT activity in culture supernatants.

**Results:**

CIITA was expressed only in HLA-II-positive U937 *Minus* cells, and this was strictly correlated with inhibition of Tat-dependent HIV-1 LTR transactivation in *Minus* but not in *Plus* cells. Overexpression of CIITA in *Plus* cells restored the suppression of Tat transactivation, confirming the inhibitory role of CIITA. Importantly, HIV-1 replication was significantly reduced in *Plus*-CIITA cells with respect to *Plus* parental cells. This effect was independent of TRIM22 as CIITA did not induce TRIM22 expression in *Plus*-CIITA cells.

**Conclusions:**

U937 *Plus* and *Minus* cells represent an interesting model to study the role of CIITA in HIV-1 restriction in the monocytic/macrophage cell lineage. The differential expression of CIITA in CIITA-negative *Plus* and CIITA-positive *Minus* cells correlated with their capacity to support or not HIV-1 replication, respectively. In *Minus* cells CIITA targeted the viral transactivator Tat to inhibit HIV-1 replication. The generation of *Plus*-CIITA cells was instrumental to demonstrate the specific contribution of CIITA in terms of inhibition of Tat activity and HIV-1 restriction, independently from other cellular factors, including TRIM22. Thus, CIITA acts as a general restriction factor against HIV-1 not only in T cells but also in myeloid cells.

## Background

The human immunodeficiency virus-1 (HIV-1) is a devastating retrovirus that causes a severe immunologic deficiency known as acquired immunodeficiency syndrome (AIDS) [1]. HIV-1 eradication represents a current topic and a long-term challenge in the study of human viral infection [2]. HIV-1 infection causes a dramatic impairment of the host immune system mainly characterized by the depletion of CD4+ T helper (Th) cells [3]. Moreover, soon after infection discrete cell reservoirs including T cells, monocytes and macrophages are formed, in which the virus is latent or replicates at very low levels. These reservoirs are resistant even to combination anti-retroviral therapy (cART) and are responsible for the ignition of viral replication and disease progression at therapy suspension [4]. Myeloid cells such as dendritic cells (DC) and tissue macrophages play a crucial role in HIV-1 primary infection by capturing the virus through mucosal transmission [5] and subsequently by promoting virus dissemination to Th lymphocytes [6]. In addition, macrophages are resistant to the cytopathic effects of the virus, have a long lifespan and can disseminate the virus for long periods of time [7]. In peripheral blood, among the different populations of monocytes, the CD14+ CD16+ subset, which normally represents 10 % of total monocytes, is more susceptible to HIV-infection ex vivo and can increase up to 50 % in HIV-1 infected subjects in comparison to CD14+ CD16− monocytes that are dominant in healthy conditions [8]. Thus, the CD14+ CD16+ monocytes play a key role in virus dissemination and may contribute to establishing HIV-1 reservoirs in various sites including the brain [9]. The susceptibility to HIV-1 infection of myeloid cells strongly depends on their differentiation stage. Circulating monocytes, although being CD4 and CCR5 positive, are less permissive to the virus than their differentiated macrophage counterpart, likely because of high levels of basal expression of restriction factors such as apolipoprotein B mRNA editing enzyme, catalytic polypeptide-like A (APOBEC3A) [10, 11]. In this regard, many restriction factors have been discovered in last decade which inhibit different steps of the viral life cycle from capsid uncoating to viral budding. Of note is the fact that most of these factors are inducible by interferons, including interferon gamma (IFN-γ) [12–18]. The importance of restriction factors is highlighted by the observation that HIV-1 has evolved the so-called “accessory proteins”, such as Nef, Vif, Vpu and, in case of HIV-2, VpX, to antagonize the antiviral effects of host restriction factors [13].

In this scenario, our laboratory has previously identified a cellular protein with the unique peculiarity of combining the activation of both the adaptive and the intrinsic immune responses against viral pathogens. This factor encoded by the Air-1 locus and named CIITA (Class II Transactivator) is indeed the master transcriptional regulator of HLA (Human Leucocytes Antigens) class II genes [19–21]. It cooperates with DNA-bound complexes (RFX5/RFXAP/RFXANK and NF-YA/NF-YB/NF-YC) and cellular coactivators forming the so-called HLA-II enhanceosome on HLA-II promoters. By inducing the expression HLA-II molecules that serve as antigen presenting molecules to CD4+ Th cells, CIITA plays a pivotal role in the triggering of the adaptive immune response [22]. The expression of HLA-II molecules is cell-type specific, developmentally regulated, and strictly correlated to the expression of CIITA. The expression of CIITA and of HLA-II molecules is constitutive in B lymphocytes, myeloid DC and thymic epithelial cells. Moreover, in several cell types, IFN-γ either induces or increases the transcription of CIITA, which, in turn, activates HLA-II gene expression [23, 24]. We have previously demonstrated that CIITA has an intrinsic anti-viral activity. CIITA inhibits the replication of HIV-1 in human T cells by competing with the viral transactivator Tat for the binding to the Cyclin T1 subunit of the Positive Transcription Elongation Factor b (P-TEFb) [25]. Similarly, CIITA targets the viral transactivators Tax-1 and Tax-2 of Human T cell Lymphotropic virus type-1 and type-2 (HTLV-1 and HTLV-2), respectively, to inhibit viral replication by using different mechanisms [26–30]. Recently, we have demonstrated that CIITA inhibits also the persistent activation of the NF-kB pathway by Tax-1, thus, counteracting one of the major strategies used by HTLV-1 to induce T cell transformation [31]. Here, we investigated whether the inhibitory function of CIITA on Tat transactivation and, thus, HIV-1 replication occurs also in cells of the monocyte/macrophage cell lineage. To this aim we took advantage of two established U937 promonocytic cells clones characterized by distinct functional and phenotypic characteristics. The clones, named U937 clone 10 *Plus* and U937 clone 34 *Minus* (defined thereafter U937 *Plus* and U937 *Minus*, respectively) have been shown to be either permissive or non-permissive to HIV-1 replication, respectively [32]. Interestingly, a switch from non-permissive to permissive HIV-1 phenotype of U937 *Minus* cells was induced by vitamin D3, an established differentiating agent for monocytes [33]. The two clones have been previously used for the identification of host factors contributing to their divergent susceptibility to HIV-1 expression and, among other candidates, Tripartite Motif 22 (TRIM22) was expressed exclusively in U937 *Minus* but not in U937 *Plus*. In the same study, TRIM22 was shown to inhibit basal HIV-1 transcription [34]. Recently, this effect was shown to be caused by TRIM22 interference with binding of Sp1 to its target DNA sequence just upstream of the transcription start site (+1) [35].

Here, we demonstrate that the *Plus* and *Minus* U937 cell clones differ for the expression of all HLA-II loci and that this correlates with the different expression of CIITA. The HLA-II positive *Minus* cells express CIITA, whereas HLA-II negative *Plus* cells do not. More importantly, CIITA was found to be instrumental for the inhibition of HIV-1 replication as U937 *Plus* cells stably transfected with CIITA (*Plus*-CIITA) showed a significantly reduced capacity of supporting virus replication. These effects were not justified by an upregulation of TRIM22 expression in *Plus*-CIITA cells and are, therefore, independent of this restriction factor. Thus, CIITA should be considered as an HIV-1 restriction factor also in cells of the myeloid lineage in addition to CD4+ T lymphocytes.

## Methods

### Plasmids

Plasmids expressing myc epitope-tagged CIITA full-length (1–1130) (pcmCIITA), flag epitope-tagged CIITA full-length (pcfCIITA), CIITA mutants (1–321, 322–1130, 1–285, 1–252, 253–410, 1–410, 64–285) and phRL-CMV vector were previously described [27, 28, 36]. To generate pcTat expression vector, two exons-Tat cDNA was amplified from pRPneo-c/SL3-Tat with the following primers: forward 5′atatatgaattcatggagccagtagatcct 3′; reverse 5′atatatctcgagctattccttcgggcctgt 3′. The PCR amplified product was digested with EcoRI and XhoI enzymes and inserted in EcoRI/XhoI digested pcDNA3 vector [37]. The reporter plasmid pHIV-1 LTRLuc vector was a gift of Prof. M. B. Peterlin (UCSF).

### Cell cultures and generation of U937 *Plus* cells stably expressing CIITA

Human embryonic kidney 293T cells were maintained in DMEM medium. The monocytic U937 *Plus* and *Minus* cells and the Raji B cell line were grown in RPMI-1640 medium supplemented with 10 % heat-inactivated fetal calf serum and 5 mM l-glutamine. U937 *Plus* cells were transfected with 5 μg of pcfCIITA plasmid by electroporation with the GenePulser II apparatus (Bio-Rad, Hercules, CA) at 300 V and 250 μF. Transfected U937 *Plus*-CIITA cells underwent G418 selection 1 mg/ml (Sigma Chemical Corp., St. Louis, MO). HLA-II DR-positive cells were enriched by fluorescence-activated cell sorting with a BD FACS ARIA II cell sorter (Becton–Dickinson, Franklin Lakes, NJ) and subjected to limiting-dilution cloning.

### Antibodies

The following monoclonal antibodies (mAb) were used for HLA staining: anti-HLA class I (B9.12.1), anti-HLA class II DR (D1.12), anti-HLA class II DP (B7/21), anti-HLA class II DQ1 (BT/3.4), DQ2 (XIII358.4), and DQ3 (XIV466.2). Anti-DQ mAb used recognize each multiple DQ alleles, and all together cover the entire set of described DQ alleles [38]. As secondary antibodies, either a FITC-conjugated anti mouse F(ab)_2_ (Sigma), or FITC-labeled IgG2a antibodies were used. Anti-flag-M2 mAb and anti-tubulin polyclonal antibody were purchased from Sigma. Anti-CIITA (7-1H) mAb was from Santa Cruz Biotechnology (Santa Cruz, CA).

### Transient transfection and Luciferase (Luc) assay

For Luciferase gene reporter assays, U937 cells were seeded in 6 well plates (2 × 10^6^ cells/well) and transfected with 0.6 μg of pHIV-1 LTRLuc, 0.15 μg of phRL-CMV plasmids and increasing amounts of pcTat, by using FugeneHD (Promega Madison,WI). The empty pcDNA3.1 vector was used as a stuffer DNA to maintain constant the total amount of transfected DNA. Cell extracts were prepared 48 h post-transfection and assayed for Luc activities by using the dual Luc reporter assay system (Promega) according to the manufacturer’s instructions. Mean Luc values, normalized to the Renilla values, of at least three independent experiments performed in duplicate were expressed as Tat-dependent vs basal fold of activation. Error bars represent the standard deviation.

### Immunoprecipitation

CIITA protein was precipitated from 60 × 10^6^ U937 *Minus* and *Plus* cells and from 30 × 10^6^ U937 *Plus*-CIITA and Raji B cells. Cells were lysed on ice for 45 min with lysis buffer (1 % NP-40, 10 mM Tris–HCl pH 7.4, 150 mM NaCl, 2 mM EDTA) supplemented with 0,1 % protease inhibitor cocktail (Sigma). After pre-clearing with protein A-Sepharose beads, whole cell extracts were incubated with anti-CIITA mAb for 1 h and immunoprecipitated with protein A-Sepharose beads overnight at 4 °C. The precipitated proteins were resolved on 8 % SDS-PAGE and analyzed by Western blotting with the anti-CIITA mAb.

### FACS analysis

The cells were collected, washed with PBS and then incubated with the anti-HLA antibodies for 30 min on ice. After washing, the cells were incubated with FITC-labeled anti-mouse Ab for 30 min on ice. The cell surface expression of HLA molecules was analyzed with an EPICS XL flow cytometer (Beckman Coulter, Pasadena, CA) and results were analyzed by the EXPO32 software (Beckman Coulter).

### Quantification of mRNA by real-time PCR (qRT-PCR)

Total RNA, deriving from 2–3 independent experiments, was extracted from cells using TRIzol reagent (Thermo Fisher Scientific, Waltham, MA). cDNA was synthesized from 2 μg total RNA using oligo(dT) primers and M-MLV reverse transcriptase (Promega). One microgram of cDNA was amplified by PCR by using an ABI Prism 7000 sequence detection system (Thermo Fisher Scientific) with IQSYBR Green PCR master mix (Bio-Rad, Hercules, CA) according to the manufacture’s protocol. Each reaction was performed in triplicate. All mRNA values were normalized to Ribosomal Protein S7 (RPS7) mRNA. The following primer pair sets were used: CIITA forward 5′-cctgctgttcgggacctaaaa-3′; reverse primer, 5′-ggatccgcaccagtttgg-3′; TRIM22, forward 5′-cactcttctcccctgattcaa-3′, reverse 5′-tcacaaactcctgcagtgct-3′; HLA-II DR, forward 5′-ctcttctcaagcactgggagttt-3′, reverse 5′-atgaagatggtcccaataatgatg-3′; RPS7, forward 5′-tggagatgaactcggacctc-3′, reverse 5′-cgaccaccaccaacttcaa-3′.

### Infection and quantitative PCR analysis of HIV-1 proviral DNA

U937 cells were exposed to the laboratory-adapted CXCR4-dependent HIV-1_IIIB/LAI_ virus at the multiplicity of infection (moi) of 1. After 1 h of virus adsorption at 37 °C, the cells were resuspended in complete medium and seeded at 5 × 10^5^ cells/well in triplicate in 96-well tissue culture plates. Culture supernatants were harvested up to 41 days and stored at −80 °C until tested for Mg2+ -dependent reverse transcriptase (RT) activity assay, as previously described [34]. Total HIV-1 DNA was quantified by qRT-PCR with an ABI 7700 prism instrument (Thermo Fisher Scientific), using primers and a probe that recognize the HIV-1 *gag* gene: forward 5′-acatcaagccatgcaaat-3′; reverse 5′-atctggcctggtgcaatagg-3′; and probe 5′-(FAM) catcaatgaggaagctgcagaatgggataga (TAMRA)-3′. The number of HIV-1 DNA copies was normalized to that of human GAPDH by an external standard curve showing a linear distribution (r = 0.99) between 10 and 10^6^ copies. The primers and probe for GAPDH were: forward 5′-accacagtccatgcatcact-3′; reverse 5′-ggccatcacgccacagtt-3′; and probe, 5′-(FAM) cccagaagactgtggatggcccc (TAMRA)-3′.

### Statistical analysis

A statistical analysis was performed using the GraphPad Prism software v. 6.0 (GraphPad Software, http://www.graphpad.com). Comparison between two groups was performed by using the unpaired *t* test. P values <0.05 were considered significant.

## Results

### Lack of CIITA expression is responsible for the HLA-II-negative phenotype of U937 *Plus* cells

**Fig. 1:**
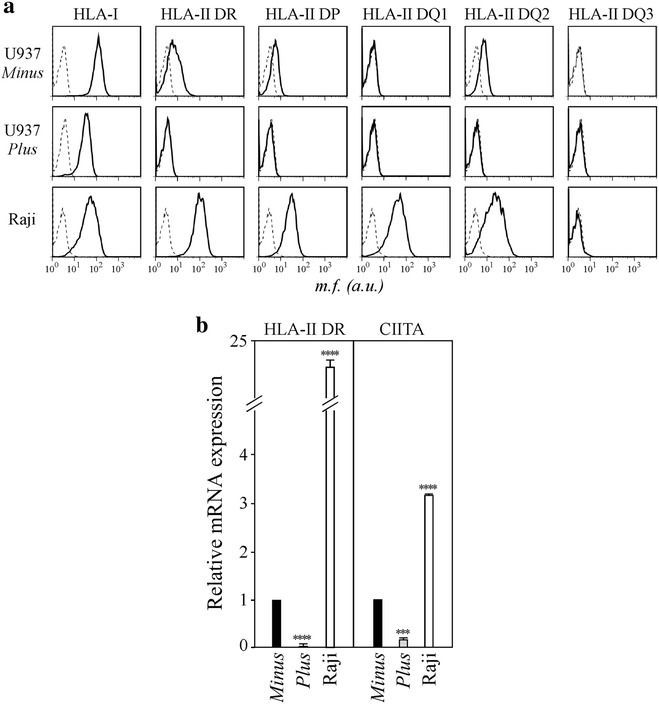
U937 *Minus* cells, but not U937 *Plus* cells express HLA-II and CIITA. **a** HLA-I and HLA-II phenotyping of U937 *Minus* and U937 *Plus* cells was carried out by immunofluorescence and FACS analysis. Raji B cells were used as a positive control for both HLA-I and HLA-II expression. *Histograms* represent fluorescence profiles of the cells indicated on the *left*, incubated with specific anti HLA-I or HLA-II mAbs (*solid line*) or with FITC-conjugated F(ab)2 anti mouse antibody (*dashed line*). By using three mAbs each covering subgroups (DQ1, DQ2 and DQ3) of DQ molecules we found that U937 *Minus* cells are DQ2-positive. Mean fluorescence (mf) values are expressed in the abscissa as arbitrary units (au). **b** HLA-II DR and CIITA mRNAs expression in U937 *Minus* cells, U937 *Plus* cells and Raji B cells was assessed by qRT-PCR. The results of a representative experiment performed in triplicates are shown. CIITA and HLA-II DR mRNA levels are expressed as values relative to those of *Minus* cells set to 1. Raji B cells were used as a positive control for both CIITA and HLA-II DR mRNA expression. Unpaired two-tailed t test has been performed (****P < 0.0001; ***P = 0.0001). *Error bars* represent the standard deviation

To verify that the two U937 *Plus* and *Minus* isogenic cell clones differ for the HLA-II cell surface expression, we firstly assessed the complete HLA-II phenotype by immunofluorescence staining and FACS analysis. HLA-II DR was not expressed by U937 *Plus* cells, whereas it was expressed by U937 *Minus* cells although at lower levels compared to Raji B cell line (Fig. 1a). Similarly, HLA-II DP and HLA-II DQ2 were expressed in U937 *Minus* cells but not in U937 *Plus* cells. Conversely, both U937 cell clones expressed HLA class-I molecules on their cell surface (Fig. 1a). To verify whether the lack of HLA-II molecules in U937 *Plus* cells was due to a transcriptional defect, we measured the amount of HLA-II DR mRNA by qRT-PCR. According to the expression of HLA-II DR molecules, we detected HLA-II DR mRNA in *Minus* but not in *Plus* U937 cells (Fig. 1b). Thus, we concluded that the complete set of HLA-II molecules was not expressed on the surface of U937 *Plus* cells consequently to a block in HLA-II genes transcription. As HLA-II expression is regulated at transcriptional level by several factors, but is strictly dependent on the presence of CIITA, we next investigated whether the different HLA-II phenotype of the two U937 clones correlated with a different expression of CIITA. To this aim, we quantified CIITA mRNA levels in both U937 clones by qRT-PCR and found that only U937 *Minus* cells expressed CIITA mRNA, whereas U937 *Plus* cells did not (Fig. 1b). Overall, these data demonstrate that CIITA expression or lack of it marks a clear-cut distinction between U937 *Minus* (CIITA-positive) and U937 *Plus* (CIITA-negative) cells. In addition, these results indicated that this cellular model represented by isogenic cell clones naturally differing for CIITA expression is suitable to define the anti-viral role of this molecule in HIV-1 infection of myeloid cells.

### Generation of U937 *Plus* cells stably expressing CIITA

**Fig. 2:**
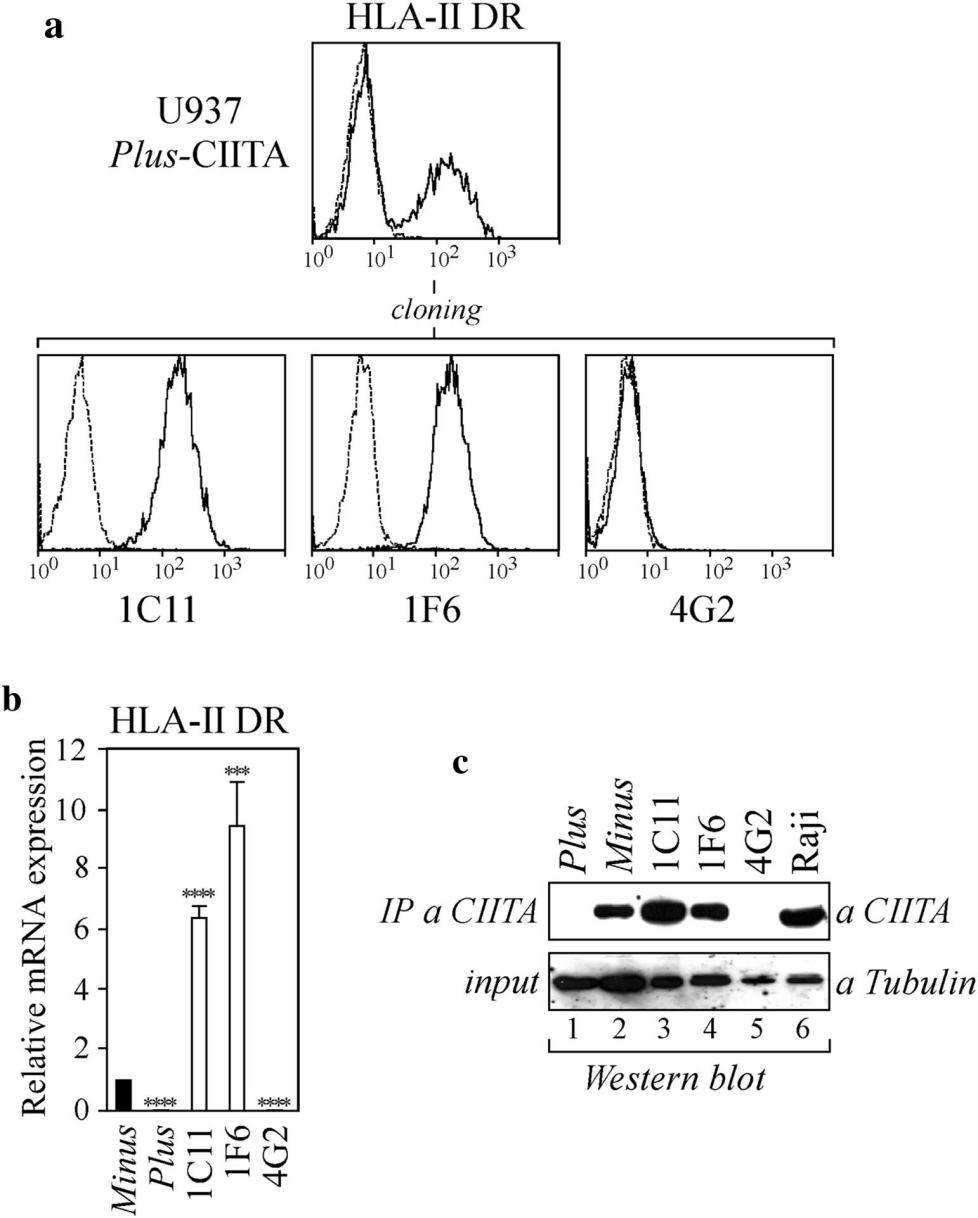
Cellular and molecular characterization of U937 *Plus* cells stably expressing exogenous CIITA. **a** U937 *Plus* cells were stably transfected with pcfCIITA vector expressing flag-tagged CIITA, generating *Plus*-CIITA cells. Bulk population was analyzed for HLA-II DR expression by FACS analysis. HLA-II-positive clones were isolated by sorting and cloning. FITC-conjugated isotype-matched secondary antibody (IgG2a) (*dotted line*) represents the negative control. **b** HLA-II DR mRNA expression was assessed by qRT-PCR in U937 *Minus* cells, U937 *Plus* cells and in clones 1C11, 1F6, 4G2. The results of a representative experiment performed in triplicates are shown. mRNA values are expressed relatively to that of *Minus* cells set to 1. Unpaired two-tailed t test has been performed (****P < 0.0001; ***P < 0.005). *Error bars* represent the standard deviation. **c** Cell lysates obtained from U937 *Plus* cells (60 × 106 cells) (*lane 1*), U937 *Minus* cells (60 × 106 cells) (*lane 2*) and CIITA-transfected *Plus* clones (30 × 106 cells) (*lanes 3–5*) were immunoprecipitated with an anti-CIITA antibody (IP a CIITA) and analyzed for the presence of CIITA by western blotting. Raji cell lysate (30 × 106 cells) was used as a positive control of CIITA expression (*lane 6*). Eight percent of the pre-cleared cell lysates was analyzed for the expression of α-tubulin by western blotting (input)

Although U937 cell clones differ for the expression of CIITA, we cannot exclude that other factors could contribute to their divergent capacity to sustain productive HIV-1 infection. Thus, to clarify the role of CIITA in HIV-1 restriction we generated U937 *Plus* cells expressing exogenous CIITA. The expression vector of flag epitope-tagged CIITA was stably transfected in the CIITA-negative U937 *Plus* parental cells. Transfected cells were selected with antibiotics and analyzed by immunofluorescence and cytofluorometry for HLA-II DR expression. A large proportion of cells expressed HLA-DR, demonstrating that CIITA alone was able to induce the expression of HLA-II genes (Fig. 2a). Highly expressing HLA-II DR cells were isolated by limiting dilution cloning and two HLA-II DR-positive clones (1C11, 1F6) and one negative clone (4G2) were selected for subsequent analyses (Fig. 2a). According to their HLA-II DR cell surface expression, the U937 cell clones 1C11 and 1F6 expressed higher levels of HLA-II DR mRNA compared to U937 *Minus* cells.

Similarly to U937 *Plus* cells, the negative clone 4G2 did not express DR mRNA (Fig. 2b).

Finally, the levels of CIITA protein expression in 1C11 and 1F6 cell clones were comparable to those of the control Raji B cell line as determined by immunoprecipitation and Western blotting (Fig. 2c, lanes 3–4 vs lane 6, respectively). The expression of CIITA in *Plus*-CIITA cells was higher than that of U937 *Minus* cells (Fig. 2c, lanes 3–4 vs lane 2) because twice the amount of U937 *Minus* cell lysate was needed to detect levels of CIITA similar to those found in clones 1C11 and 1F6 (Fig. 2c, lanes 3–4 vs lane 2). As expected, CIITA was not detected in the parental U937 *Plus* cells (Fig. 2c, lane 1) and in the HLA-II-negative 4G2 clone (Fig. 2c, lane 5). Thus, CIITA-positive U937 *Plus* cells represent an important tool to uncouple the role of CIITA from other factors which may also contribute to HIV-1 restriction in monocytic cells.

### Tat-mediated HIV-1 LTR transactivation occurs in U937 *Plus* cells but is suppressed in U937 *Minus* cells and in U937 *Plus*-CIITA clones

**Fig. 3:**
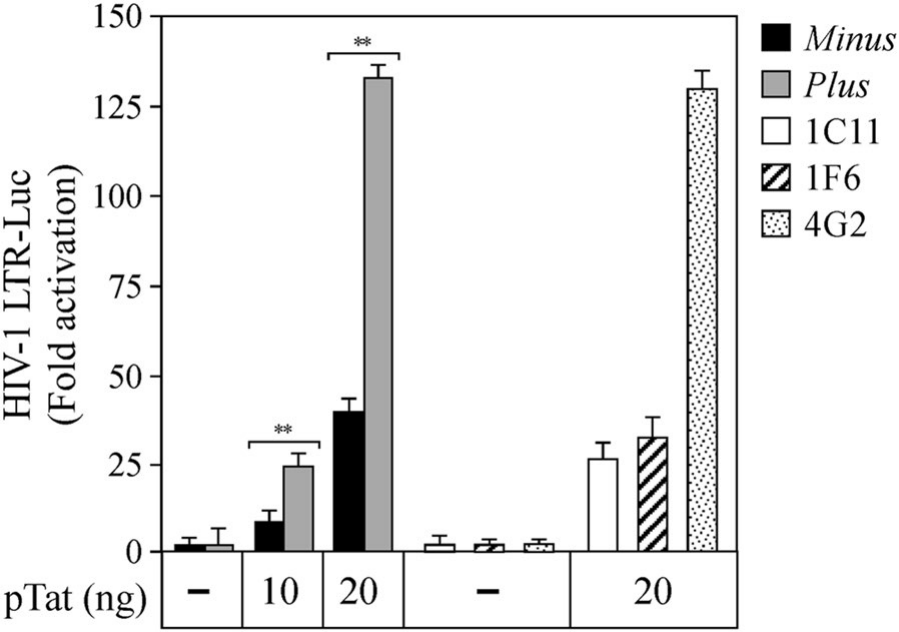
CIITA inhibits Tat transactivating function in U937 *Minus* cells and in U937 *Plus*-CIITA clones. U937 *Minus* (*black columns*) and U937 *Plus* (*gray columns*) cells were co-transfected with fixed amount of pHIV-1 LTR-Luc, phRL-CMV, and increasing amounts (10, 20 ng) of plasmid coding for Tat (pcTat). CIITA-positive *Plus* clones 1C11, 1F6 and CIITA-negative *Plus* 4G2 clone were co-transfected with fixed amount of pHIV-1 LTR-Luc, phRL-CMV and 20 ng of pcTat. The basal HIV-1 LTR activity was set to 1 for each cell (−). Mean luciferase values, normalized to Renilla values, are presented as fold of activation (Tat-dependent vs basal). Results are the average of two independent experiments (**P < 0.005, unpaired two-tailed t test). *Error bars* show standard deviation

We have previously demonstrated that CIITA inhibits HIV-1 replication in human T cells by interfering with Tat function [25]. Therefore, we investigated whether the HIV-1 permissive and non-permissive phenotypes of U937 *Plus* and *Minus* cells, respectively, were due to their capacity to support or inhibit Tat-dependent HIV-1 LTR transactivation as a consequence of their different expression of CIITA. To this aim, we measured the Tat-dependent luciferase activities in U937 *Minus*, *Plus* and *Plus*-CIITA cells transiently transfected with the pHIV-1LTR-Luc reporter gene and increasing amounts of Tat expressing vector. An efficient Tat-dependent transactivation of HIV-1 transcription was observed in U937 *Plus* cells, whereas it was significantly reduced in the U937 *Minus* cells at both doses of Tat (Fig. 3, grey and black columns, respectively). Remarkably, in U937 *Plus*-CIITA clones 1C11 and 1F6 transfected with the highest amount of Tat plasmid, Tat transactivation is inhibited similarly to U937 *Minus* cells. In the 4G2 cell clone devoid of CIITA expression, Tat-mediated HIV-1 LTR transactivation was unaffected (Fig. 3). These results indicate that the permissive and the non-permissive phenotypes to HIV-1 infection of U937 *Plus* and *Minus* cells correlate with their capacity or incapacity, respectively, to support Tat-mediated HIV-1 LTR transcription. Importantly, the stable expression of CIITA in *Plus* cells abrogated Tat transactivating function. Thus, CIITA plays a major role in the inhibition of Tat transactivation, not only in T cells but also in myeloid cells, at least in the cellular model here investigated.

### CIITA region 64–285 is required for the inhibition of Tat-dependent activation of the HIV-1 LTR

**Fig. 4:**
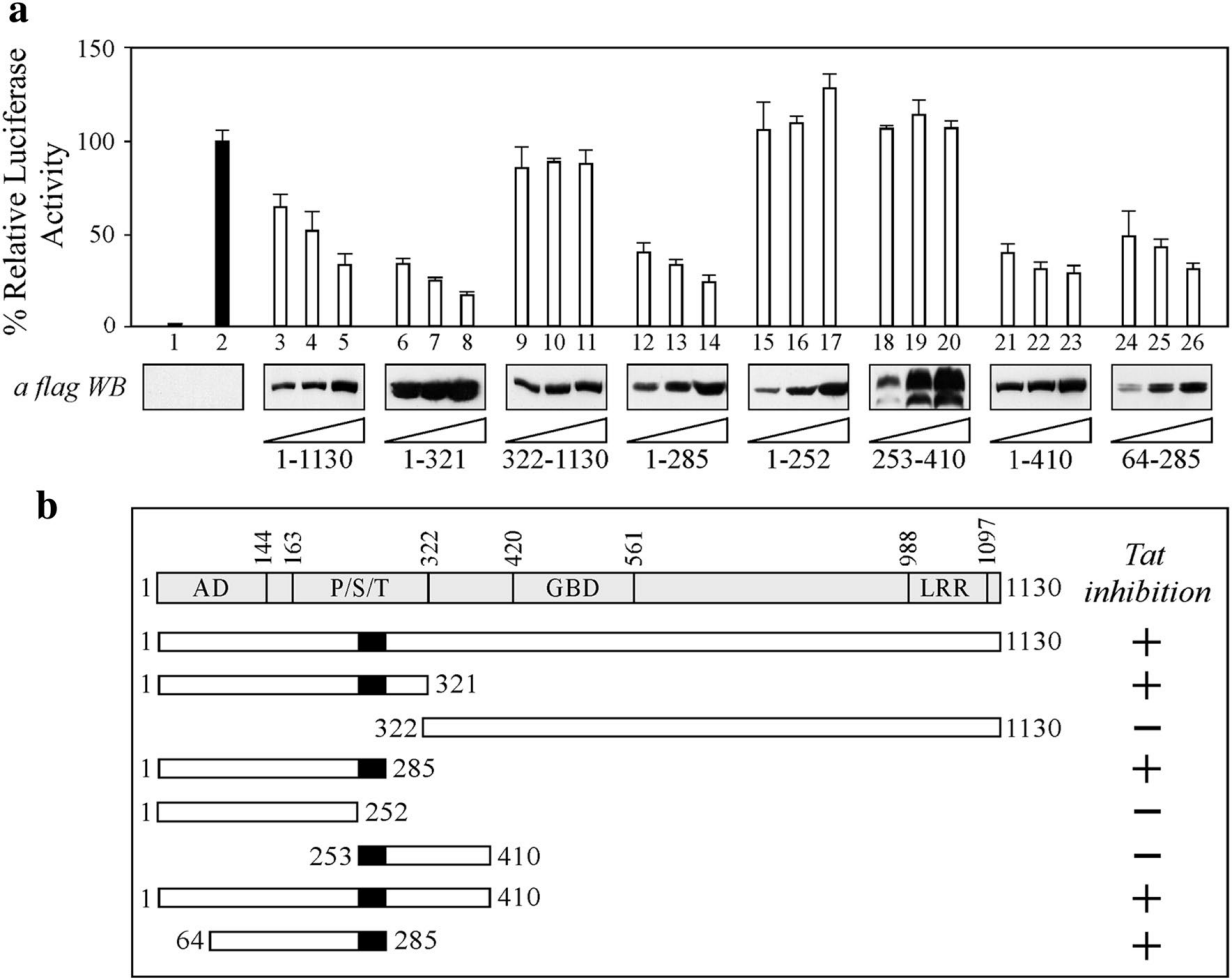
Residues 253-285 of CIITA are essential but not sufficient to inhibit Tat-mediated HIV-1 LTR transactivation. **a** 293T cells were co-transfected with fixed amounts of pHIV-1LTR Luc, phRL-CMV, pTat and increasing amounts of flag-tagged CIITA full-length or the deletion mutants listed below the western blot analyses. The results of a representative experiment are shown. Mean luciferase activities, normalized to Renilla activity, are presented as percentages relative to activation by Tat set to 100 % (*black column 2*). *Column 1* represents the basal activity of cells transfected with the pcDNA3 vector. The expression of recombinant fCIITA proteins in cell extracts was evaluated by anti-flag western blotting. **b** Schematic representation of the results of the gene reported assay illustrated in *panel* **a**. CIITA proteins used for the mapping are shown, along with their capacity to inhibit Tat-dependent activation of LTR promoter (+). The endpoints of CIITA full-length and CIITA deletion mutants are indicated. At the top is a diagram of CIITA with its domains, labeled as follows: *AD* activation domain, *P/S/T* proline/serine/threonine-rich domain, *GBD* GTP-binding domain, and *LRR* leucine-rich repeats

CIITA is a protein composed of 1130 amino acids, containing several functional domains regulating its biological activity. To define the region(s) of CIITA responsible for inhibiting the activation of the HIV-1 promoter by Tat, several deletion mutants of CIITA were expressed in 293T cells and analyzed for their ability to suppress Tat-promoted HIV-1 LTR-dependent luciferase activities in comparison to full-length CIITA. Confirming the results obtained in CIITA-positive U937 cells, CIITA full-length inhibited Tat activity in a dose-dependent manner (Fig. 4a, lanes 3–5 vs lane 2). By using two complementary CIITA fragments, 1-321 and 322–1130, we determined that the N-terminal 1–321, but not the C-terminal 322–1130 deletion mutant, inhibited Tat activity (Fig. 4a, lanes 6–8 and lanes 9–11, respectively). Thus, we focused on the N-terminal part of CIITA to restrict the region mediating its inhibitory effect. The mutant encompassing amino acids 1–285 still inhibited Tat function, similarly to CIITA 1–321 (Fig. 4a, lanes 12–14 vs lane 2). On the contrary, CIITA 1-252 did not exert any suppressive effect (Fig. 4a, lanes 15–17 vs lane 2) indicating that residues 253–285 were crucial for the inhibition of Tat function (Fig. 4b, black box). Surprisingly enough, the CIITA fragment 253–410, containing the inhibitory domain, did not block LTR transactivation by Tat (Fig. 4a, lanes 18–20 vs lane 2), suggesting that the inhibitory domain 253–285 must be extended at the N-terminus in order to accomplish its suppressive effect. Indeed, the extension of fragment 253–410 to residue 1 rescued the ability to inhibit Tat by CIITA 1-410 (Fig. 4a, lanes 21–23 vs lane 2). Of interest, the deletion of the first 63 amino acids in CIITA 1–285 did not abrogate its capacity to suppress Tat activity (Fig. 4a, lanes 24–26 vs lane 2). Overall, these results show that the CIITA region encompassing residues 64–285 is the minimal domain required for the inhibition of Tat-dependent LTR transactivation.

### HIV-1 replication is inhibited in U937 *Plus*-CIITA cells

**Fig. 5:**
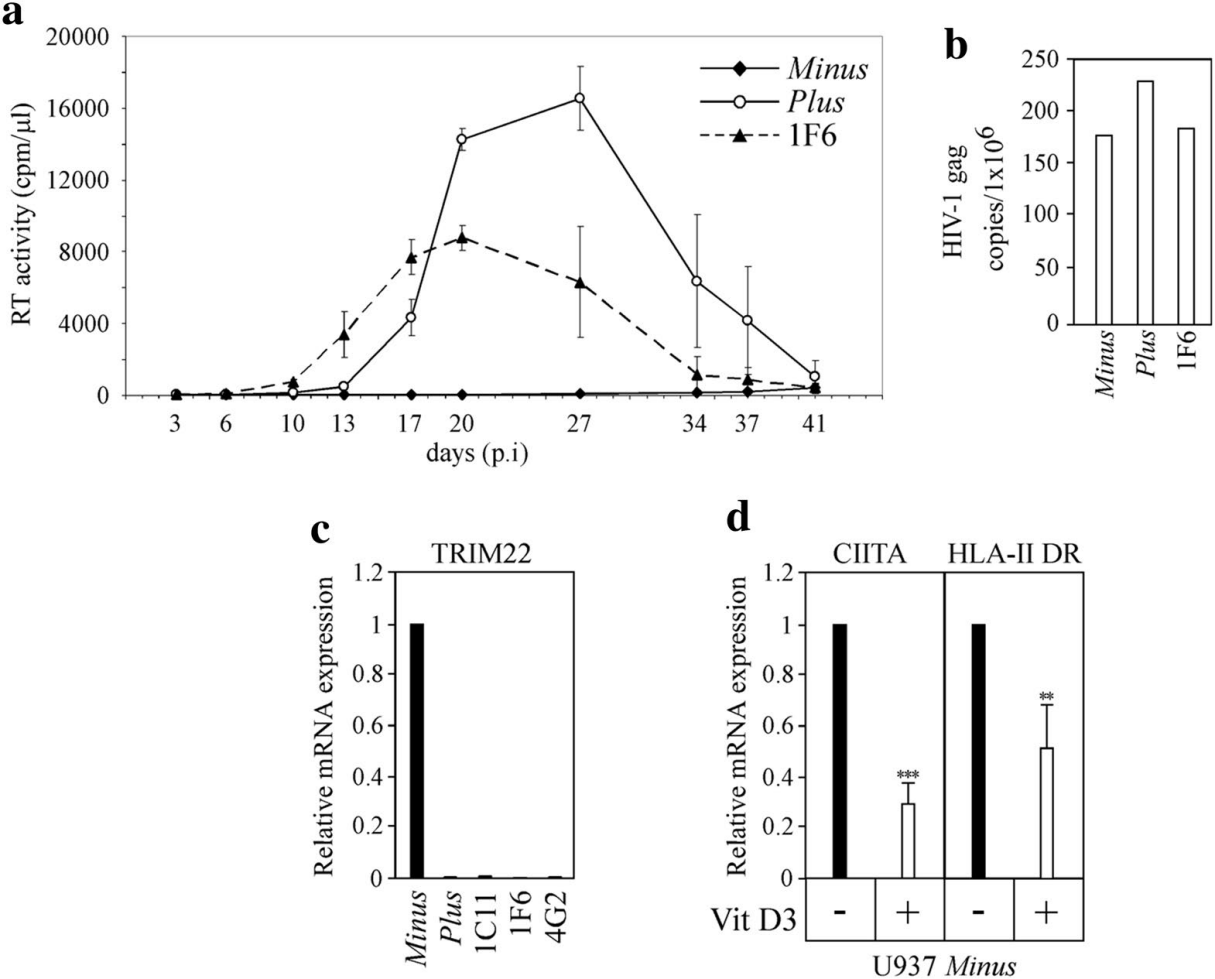
CIITA stably expressed in U937 *Plus* cells inhibits HIV-1 replication without inducing TRIM22 transcription. **a** U937 *Minus* cells, U937 *Plus* cells and U937 *Plus*-CIITA 1F6 clone were infected with HIV-1_IIIB/LAI_ and viral replication was measured by monitoring RT activity in the culture supernatants up to 41 days post-infection (pi) Mean values of triplicate cultures are shown. The data are representative of four independent experiments. **b** At 24 h pi the amount of HIV-1 proviral DNA was assessed by real time PCR by using primers specific for gag. **c** TRIM22 mRNA expression was assessed by qRT-PCR in U937 *Minus* cells, U937 *Plus* cells and in clones 1C11, 1F6, 4G2. U937 *Minus* cells TRIM22 mRNA level was set to 1. **d** U937 *Minus* cells either untreated (−) or treated (+) with 10 nM vitamin D3 for 72 h were analyzed for CIITA and HLA-II DR mRNAs expression by qRT-PCR. In Vitamin D3-untreated U937 *Minus* cells the amount of both CIITA and HLA-II DR mRNAs was set to 1. Results are the average of three independent experiments (***P = 0.0001; **P = 0.0094, unpaired two-tailed t test). *Error bars* show standard deviation

To assess whether the CIITA-mediated inhibition of Tat function observed in CIITA-positive U937 cells was correlated with an inhibition of HIV-1 replication we infected the different U937 clones with HIV-1_IIIB/LAI_ and measured the levels of RT viral activity in the culture supernatants up to 41 days post-infection. As previously shown [32, 34], HIV-1 replicates efficiently in U937 *Plus* cells reaching a peak at day 27 (Fig. 5a, solid lane, open circle). In contrast, virus replication in U937 *Minus* cells was not detected in this time frame (Fig. 5a, solid lane, solid square), consistently with previous publications [34]. Of note, in CIITA-expressing U937 *Plus* clone 1F6 the HIV-1 replication was significantly inhibited with respect to U937 *Plus* cells (Fig. 5a, dashed lane, solid triangle). In particular, at day 27 the RT activity measured in U937 Plus-CIITA 1F6 clone was less than 40 % the one measured in U937 *Plus* cells (6.318 and 16.534 cpm/μl, respectively). This different profile of virus replication was not accounted for by a different infection efficiency in that the amount of HIV-1 DNA detected 24 h post-infection was comparable in all tested cells (Fig. 5b). In conclusion, the results obtained by infecting U937 *Plus*-CIITA clone 1F6, indicate that CIITA contributes, at least in part, in determining the non-permissive phenotype of U937 *Minus* cells by inhibiting Tat activity. In U937 *Plus*-CIITA 1F6 clone, HIV-1 replication was significantly suppressed compared with *Plus* parental cells, but did not reach the inhibition level observed in *Minus* cells (Fig. 5a). This result let us to hypothesize that other host factors with anti-viral functions may contribute to the non-permissive phenotype of *Minus* cells. Among these, TRIM22, expressed in U937 *Minus* cells, but not in U937 *Plus* cells, was shown to inhibit basal as well as PMA plus ionomycin-induced HIV-1 transcription independently of Tat and NF-kB [34]. Therefore, we verified whether CIITA transfection in *Plus* cells induced TRIM22 mRNA as quantified by qRT-PCR. We found that only U937 *Minus* cells expressed TRIM22 mRNA whereas CIITA-positive 1C11 and 1F6 clones as well as the CIITA-negative 4G2 clone did not (Fig. 5c). Thus, in CIITA-expressing U937 *Plus* clones, the impairment of HIV-1 replication is solely due to CIITA and not to the induction of TRIM22 expression.

It has been reported that the treatment of U937 *Minus* cells with 1,25 Dihydroxyvitamin D3 (Vitamin D3) converts their non-permissive phenotype in the permissive one [33]. Therefore, we assessed whether Vitamin D3 treatment was also associated with the modification of CIITA and HLA-II expression in *Minus* cells. Indeed, Vitamin D3 induced a strong down-regulation of CIITA mRNA and, by consequence, of HLA-II (Fig. 5d). These results suggest that in Vitamin D3-treated *Minus* cells, the down-regulation of CIITA expression, could contribute to the previously shown increased susceptibility to HIV-1 productive infection [33].

## Discussion

We have previously demonstrated that CIITA, the master regulator of HLA-II gene transcription, inhibits HIV-1 replication in CD4+ human T cell lines expressing exogenous CIITA. This inhibition occurs by preventing the binding of HIV-1 Tat to the Cyclin T1 subunit of the P-TEFb complex, used by the virus to elongate the viral transcripts. In this study, we used two established U937 cell clones to investigate whether CIITA inhibits HIV-1 replication also in the monocytic cells. CIITA was expressed in the HIV-1-non-permissive U937 clone 34 *Minus*, but not in the HIV-1-permissive U937 clone 10 *Plus*, and this strongly correlated with the inhibition of Tat-mediated HIV-1 LTR transactivation in *Minus* but not in *Plus* clone. To substantiate this hypothesis we have modified U937 *Plus* cells by introducing a genetic cassette expressing exogenous CIITA. The expression of CIITA in modified U937 *Plus* cells significantly reduced the Tat transactivating function in comparison to the U937 *Plus* parental cell line. This finding suggested a prominent role of CIITA in HIV-1 restriction in monocytic cells. The expression of CIITA alone significantly reduced HIV-1 replication in *Plus*-CIITA cells compared to U937 *Plus* parental cells. However, the potency of inhibition of HIV-1 replication in *Plus*-CIITA cells did not reach that observed in U937 *Minus* cells supporting the hypothesis that other factors cooperate with CIITA in determining the long lasting restriction of virus replication typical of U937 *Minus* cells.

In this regard the host restriction factor TRIM22, as CIITA, is expressed exclusively in U937 *Minus* cells and it was shown to inhibit basal as well as PMA+ ionomicyn induced HIV-1 transcription [34]. Therefore, we ruled out the possibility that CIITA transfection caused an upregulated expression of TRIM22 and concluded that these two factors act independently and, at least potentially, synergistically, in terms of inhibiting viral transcription and replication. By comparing the effect on virus replication of CIITA and TRIM22, it should be noted that the levels of inhibition in U937 *Plus* cells was comparable upon transduction of either TRIM22 [34] or CIITA and that neither factor alone could account for the extremely restricted phenotype of U937 *Minus* cell clone. Future studies aimed at identifying the possible functional relationship between these two factors will bring further insight into the molecular mechanism involved in HIV-1 restriction.

It has been shown that Vitamin D3 stimulation promotes HIV-1 replication in U937 *Minus* cells by reverting the non-permissive phenotype to a permissive one. This effect was partially attributed to an increased expression of CXCR4 and increased viral entry, although other factors have been suggested to be involved in the induction of the permissive phenotype [33]. Moreover, Vitamin D3 treatment has been shown to negatively modulate the expression of HLA-II genes in IFN-γ-treated cells [39] and, particularly, in human blood circulating monocytes [40]. Finally, it has also been shown that in Vitamin D3-treated U937 cells the expression of HLA-II was down-modulated with respect to the untreated cells [41]. The above observations did not explain the intimate mechanism at the basis of this down-modulation. Here, we demonstrated that the decreased expression of HLA-II molecules in U937 *Minus* cells after Vitamin D3 treatment is due to the transcriptional impairment of both HLA-II structural genes and their transcriptional regulator CIITA. Overall, these findings further reinforce the association between CIITA expression and the inhibition of HIV-1 replication in monocytic compartment.

CIITA is a large protein of 1130 amino acids and it was interesting to assess whether only the full-length molecule or a part of it could inhibit Tat function. We used several N-terminal and C-terminal deletion mutants of CIITA to map the region responsible for the inhibition of Tat activity. A short stretch of 32 amino acids from positions 253 to 285 was strictly required for the inhibitory effect of CIITA. Remarkably, this amino acid sequence needed to be extended at the N-terminus to exert the inhibitory function. It has been shown that Cyclin T1 of the P-TEFb complex binds to the first 322 amino acids of CIITA [42]. Thus, it is possible that the extension to the N-terminus of the inhibitory domain identified by our functional mapping, is necessary for a correct folding of CIITA, in order to bind Cyclin T1. Interestingly enough, this region does not correspond to that involved in the inhibition of HTLV-1 Tax-1- and HTLV-2 Tax-2-mediated LTR transactivation [27, 28], indicating that CIITA exploits different mechanisms for the inhibition of the different retroviral transactivators.

Although macrophages and CD4+ T cells are both major targets of HIV-1, the susceptibility to viral infection is different in the two cell types and depends on the activation status of the cells [43, 44]. Multiple cellular factors may be limiting for productive HIV-1 infection in these cells. Among these the P-TEFb complex, resulting from Cyclin T1 and CDK9 heterodimeric association, is essential for Tat function [42]. P-TEFb is differentially expressed in CD4+ T lymphocytes and monocytes/macrophages, depending on their activation and differentiation status [45]. Therefore, it is conceivable that the inhibition of Tat activity by CIITA may be influenced not only by the squelching of Cyclin T1 by CIITA but also by the modulation of Cyclin T1 expression itself in myeloid cells. Within this frame, it has been shown that PMA treatment, a stimulus inducing the differentiation of U937 promonocytic cells into macrophage-like cells results in increased levels of HIV-1 replication and Cyclin T1 expression [46]. Remarkably, we previously showed that in PMA-differentiated U937 cells, the mRNA levels of both HLA-II and CIITA are strongly down-regulated with respect to control unstimulated cells [47]. Thus, the increased susceptibility to HIV-1 productive infection in PMA-treated U937 cells may correlate both with the increase of Cyclin T1 and with the down-regulation of CIITA restriction factor.

## Conclusions

In conclusion our findings confirm and extend the knowledge on the role of CIITA acting as a restriction factor for HIV-1. Unlike other host restriction factors, CIITA has the peculiar characteristic of both activating the adaptive immune response to pathogens and suppressing viral gene expression by targeting the activity of the viral transactivators. This dual function of CIITA against retroviruses operates not only in T cells [25, 48], but also in the monocyte-macrophage cell lineage, endowed with both adaptive and intrinsic immune functions.

## Abbreviations

CIITA: class II transactivator
HLA: human leucocytes antigens
P-TEFb: positive transcription elongation factor b
HIV-1: human immunodeficiency virus-1
Th: T helper cells
TRIM22: tripartite motif 22
IFN-γ: interferon gamma
Vitamin D3: 1,25 dihydroxyvitamin D3
LTR: long termimal repeat
FACS: fluorescence activated cell sorter
